# A super-stable homogeneous Lipiodol-hydrophilic chemodrug formulation for treatment of hepatocellular carcinoma

**DOI:** 10.7150/thno.68456

**Published:** 2022-01-24

**Authors:** Pan He, En Ren, Biaoqi Chen, Hu Chen, Hongwei Cheng, Xing Gao, Xiaoliu Liang, Hao Liu, Jingdong Li, Bo Li, Aizheng Chen, Chengchao Chu, Xiaoyuan Chen, Jingsong Mao, Yang Zhang, Gang Liu

**Affiliations:** 1 State Key Laboratory of Molecular Vaccinology and Molecular Diagnostics, Center for Molecular Imaging and Translational Medicine, School of Public Health, Xiamen University, Xiamen 361102, China.; 2 Department of Radiology, Xiang'an Hospital of Xiamen University, School of Medicine, Xiamen University, Xiamen 361102, China.; 3 Fujian Provincial Key Laboratory of Biochemical Technology, Institute of Biomaterials and Tissue Engineering, Huaqiao University, Xiamen 361021, China.; 4 Amoy Hopeful Biotechnology Co., Ltd., Xiamen 361027, China.; 5 Department of General Surgery, Institute of Hepatobiliary-Pancreatic-Intestinal Diseases, Affiliated Hospital of North Sichuan Medical College, Nanchong 637000, China.; 6 Department of General Surgery (Hepatobiliary Surgery), The Affiliated Hospital of Southwest Medical University, Luzhou 646000, China.; 7 Departments of Diagnostic Radiology, Chemical and Biomolecular Engineering, and Biomedical Engineering, Yong Loo Lin School of Medicine and Faculty of Engineering, National University of Singapore, Singapore, Singapore.

**Keywords:** Hepatocellular carcinoma, Theranostics, Transcatheter arterial chemoembolization, Homogeneous formulation, Embolic agent

## Abstract

**Background:** Though lipiodol formulations are major options in transcatheter arterial chemoembolization (TACE) of advanced unresectable hepatocellular carcinoma (HCC) in the clinic, their application is severely limited by insufficient physical stability between the hydrophobic lipiodol and hydrophilic drugs; thus, most chemotherapeutic drugs are quickly released into systemic circulation resulting in poor therapeutic outcomes and serious side effects.

**Methods**: The typical hydrophilic drug doxorubicin hydrochloride (DOX) was prepared as a pure nanomedicine and then stably and homogeneously dispersed in lipiodol (SHIFT&DOX) *via* slightly ultrasonic dispersion. The drug release profiles of SHIFT&DOX were defined in a decellularized liver model. *In vivo* therapeutic studies were performed in rat-bearing N1S1 orthotopic HCC models and rabbit-bearing VX2 orthotopic HCC models.

**Results**: SHIFT&DOX features an ultrahigh homogeneous dispersibility over 21 days, which far surpassed typical Lipiodol-DOX formulations in clinical practice (less than 0.5 h). SHIFT&DOX also has excellent sustained drug release behavior to improve the local drug concentration dependence and increase the time dependence, leading to remarkable embolic and chemotherapeutic efficacy, and eminent safety in all of the orthotopic HCC models.

**Conclusions:** The carrier-free hydrophilic drug nanoparticle technology-based lipiodol formulation provides a promising approach to solve the problem of drug dispersion in TACE with the potential for a translational pipeline.

## Introduction

Hepatocellular carcinoma (HCC) is the most common type of liver cancer. It has a poor prognosis and high mortality rate that makes it the third leading cause of cancer deaths worldwide 1,2. Surgical treatment is the first choice in the clinical treatment of liver cancer 3,4. However, only 10 to 15 percent of patients with HCC are suitable for surgical resection due to a lack of early detection and rapid tumor growth 5. Systemic intravenous chemotherapy is useful for HCC treatment, but the response rate is only 20% with no significant clinical value to patient survival 6,7. More recently, transcatheter arterial chemoembolization (TACE) has become the mainstream palliative treatment for unresectable HCC with value in therapeutic efficiency 8,9. Lipiodol-based TACE is currently recognized as a mainstay of treatment for intermediate and advanced liver cancer 10,11. It is a green, economic, and patient-friendly approach due to it being specifically deposited in the tumor microenvironment, while metabolized in normal liver tissues 12,13. Nevertheless, the application of lipiodol is severely hindered in TACE due to insufficient physical stability between the hydrophobic phase and hydrophilic drugs 14-16. Many combined drugs are prepared into coarse formulations by traditional manual mixing methods with poor reproducibility and stability. They often separate within 30 minutes and lack sustained release behavior, long-term action, or low toxicity 17,18. Therefore, it is urgent to achieve stable dispersion of hydrophilic drugs in lipiodol. Fortunately, we previously proposed and validated a super-stable homogeneous intermixed formulation technology (SHIFT) that utilizes the expansibility of supercritical carbon dioxide (SC-CO_2_) fluid to realize homogeneous indocyanine green (ICG) dispersion in lipiodol 19-21 and achieve drug nanocrystalization without additional ingredients or residual solvents 22,23. We reasoned that a carrier-free hydrophilic drug nanoparticle technology-based SHIFT system could achieve stable and homogeneous dispersion of hydrophilic drugs in lipiodol to overcome the bottleneck of TACE development.

**Figure 1:**
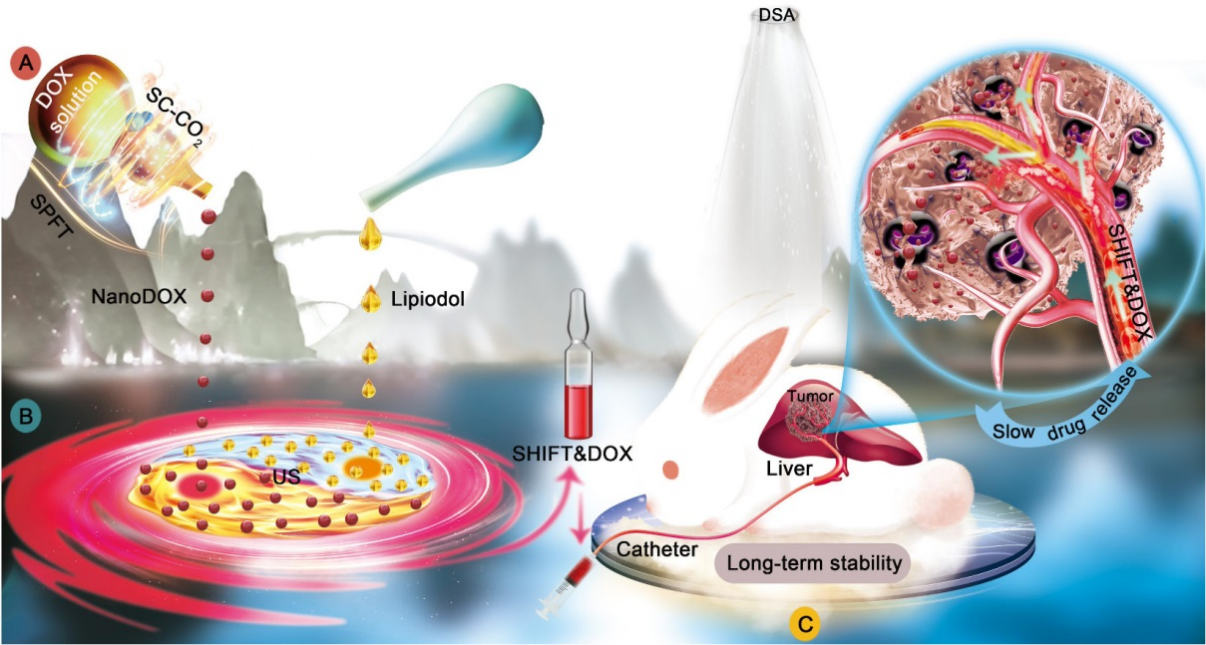
Schematic illustration of SHIFT&DOX preparation and transarterial chemoembolization of HCC. A: Superstable pure-nanomedicine formulation technology (denoted as SPFT) was used to produce nanoDOX with a smaller nanoparticle size and homogeneity. B: Subsequently, nanoDOX was homogeneously dispersed into lipiodol *via* ultrasonication (US) to prepare the SHIFT&DOX. C: The SHIFT&DOX specifically deposited into hepatocellular carcinoma lesions through transcatheter embolization. This then led to long-term DOX stability and slow drug release from lipiodol to improve treatment effects and safety of HCC.

In this study, the typical hydrophilic chemotherapeutic drug doxorubicin hydrochloride (DOX) was prepared as a pure nanomedicine and then stably and homogeneously dispersed in lipiodol (SHIFT&DOX) *via* slightly ultrasonic dispersion (Figure 1). Different from the traditional mixture of freeDOX and lipiodol, SHIFT&DOX not only has a good deposition in the tumor lesions, but is stable with excellent homogeneity over 21 days. We demonstrate that with a small particle size and regularly spherical morphology, nanoDOX has slight gravity, and increases the contact angle with hydrophobic phase, thus keeping stable in lipiodol 24,25. Furthermore, *in vitro* and *in vivo* results (*i.e.*, decellularized liver drug release models, rat-bearing N1S1 orthotopic HCC models, and rabbit-bearing VX2 orthotopic HCC models) show that SHIFT&DOX exhibited a specifically prolonged drug retention and excellent sustained drug release effect in tumor regions. Therefore, SHIFT&DOX greatly solves the terrible instability, sudden drug release, poor efficacy, unpromising reproducibility and serious side-effect issues of rough lipiodol emulsions used in the clinic.

## Materials and methods

### Materials

Lipiodol was purchased from Jiangsu Hengrui Pharmaceutical Co., Ltd. (Lianyungang, China). Doxorubicin hydrochloride was purchased from Aladdin Biochemical Technology Co., Ltd. (Shanghai, China). Pentobarbital sodium was purchased from Lulong biotechnology Co., Ltd. (Shanghai, China). Dichloromethane and ethyl alcohol were purchased from Sigma-Aldrich Chemical Co. (Saint Louis, MO, USA). The super-stable pure-nanomedicine formulation technology (SPFT) equipment was developed in our laboratory (Patent nos. 2019107349374.4; 201910105683X and 16581600), Xiamen University (Xiamen, China). Interventional medical devices were available from the Radiology Department of Xiang'an Hospital of Xiamen University.

### Synthesis and characterization of nanoDOX

A total of 40 mg of DOX was dissolved in 10 mL of methyl alcohol, and then the mixed solution was transferred to the SC-CO_2_ reactor (10 MPa, 45℃) at a speed of 1 mL/min. Fresh CO_2_ was pumped into the high pressure vessel at a constant flow rate of 35 g/min to remove the solvent, resulting in the precipitation of nanoDOX. After the solution was completely injected into the vessel, fresh CO_2_ was continued for additional 30 min to completely remove the residual solvent. Then, the collected nanoDOX was characterized by transmission electron microscopy (TEM) on a TecnaiG2 Spirit Electron Microscope (FEI Co., Hillsboro, OR, USA), and dynamic light scattering (DLS) using a Malvern Zetasizer (Malvern Instruments Ltd., Malvern, UK). The analysis of the drug structure was performed by liquid chromatography-mass spectrometry (LC-MS) using a shimadzu high-pressure liquid chromatography (HPLC) system equipped with an SPD-20A ultraviolet-visible (UV-VIS) light detector (Shimadzu., Kyoto, Japan) and scanning electron microscopy (SEM) on a Leica EM CPD300 Scanning Electron Microscope (Leica Microsystems GmbH, Wetzlar, German). The fluorescence imaging of the reporter and fluorescent signal intensity measurements were performed using an IVIS Lumina III Fluorescence Imaging system (Hopkinton, USA). The contact angle detection was performed using a DSA-100 system (Hamburg, German).

### Synthesis and characterization of SHIFT&DOX

For the preparation of SHIFT&DOX, the required amount of nanoDOX and lipiodol were simply mixed and ultrasonicated in water bath for 5 min, and the sample was designated as SHIFT&DOX. For the drug release, 300 μL SHIFT&DOX (1 mg/mL) was injected into the bottom of the 0.9% saline (600 μL), and placed in orbital shaker at 100 rpm at 37°C for 168 h (7 days). At predetermined time point (1, 2, 3, 5, and 7 days), 200 μL saline was collected from each sample and replaced with fresh 0.9% saline. The concentration of the collected sample was measured by spectrophotometry. Spectrophotometry using a Shimadzu UV-2100 spectrophotometer (Shimadzu Corp.), and TEM using a TecnaiG2 Spirit Electron Microscope (FEI Co.) were used to verify the molecular components of nanoDOX. The traditional iodinated formulation technology (TIFT) was used to prepare TIFT&DOX formulations via simple mixture with lipiodol, which was set as control group. The viscosities of lipiodol, TIFT&DOX, and SHIFT&DOX were measured with a viscosimeter at 37°C. The dispersibility of the DOX lipiodol emulsion was determined as follows, First, lipiodol was stained with coumarin, and then 1 mg of nanoDOX or freeDOX was added into 0.5 mL of lipiodol. According to the corresponding technical experimental points, the mixture solution of 2 mg/mL was prepared and observed under a confocal microscope.

### Preparation of decellularized liver and determination of drugs release

The decellularized liver was prepared by a previously reported method 26. Briefly, the rat liver was completely removed and stored at -80°C overnight. The next day, after thawing the organ, the portal vein and inferior vena cava were washed with a 5% sodium dodecyl sulfate (SDS) solution using a peristaltic pump (at 4 mL/min) for 8 - 10 h, and then rinsed with 0.9% saline. The release of drugs was determined as follows. First, nanoDOX and freeDOX were prepared into a 2 mg/mL formulation (SHIFT&DOX, TIFT&DOX) for standby. Then, the successfully prepared decellularized liver was rinsed with 0.9% saline to wash off the SDS solution, and the corresponding formulation was slowly injected into the hepatic inferior vena cava with a 1 mL syringe. Finally, a fluorescence microscope (Exposure 250 J, gain 8 - 12) was used to capture images at different time points (0, 6, 12, 24 h). The captured images were analyzed and quantified using the Image J software.

### Cell uptake and toxicity

For cell uptake detection, 1 mL of medium (containing 10^4^ HepG-2 cells) was added to the confocal dishes and cells were allowed to adhere to the dish surface overnight. According to the time point, the medium was removed, and 1 mL of medium containing 20 μg/mL of drug was added to fix the drug. After washing it once with phosphate buffered saline (PBS), 4% paraformaldehyde was added, and washed once with PBS after incubation. Then, cells were stained with 4′,6-diamidino-2-phenylindole (DAPI), washed twice with PBS after staining, and observed/imaged by confocal fluorescence microscopy. For cell cytotoxicity measurement, 100 μL of medium per well (containing 10,000 HepG-2 cells) was added to each well of a 96-well plate and cells were allowed to attached overnight. The next day, after removing the medium from each well, the experimental group wells received 100 μL of medium containing different concentrations of drugs, and the control group wells received 100 μL of medium only and the plate was incubated for 24 h. Afterwards, the medium was removed from each well, and each well was washed with PBS. Then, the appropriate volume of the cell counting kit-8 (CCK-8) assay (Dojindo Molecular Technologies, Kumamoto, Japan) reagent in medium was added to each well, the plate was incubated for 1 h, and the absorbance was measured at 450 nm. Finally, cells were stained with a crystal violet solution for 10 min and observed under a microscope.

### N1S1 rat and VX2 rabbit syngeneic orthotopic HCC models

Animal experiments were approved by the Animal Care and Use Committee (CC/ACUCC) of Xiamen University. For the rat model, Sprague Dawley (SD) rats (100 - 120 g, specific pathogen-free) were used as experimental animals in this study. To establish the liver cancer model, 0.2 mL of N1S1 cell suspension (20 × 10^6^ cells per 100 μL of PBS) was injected into the left lateral lobe of the liver of each animal. The tumor size reached 80~100 mm^3^ after 8 days. For the rabbit model, New Zealand white rabbits (2.5 - 3.0 kg) were obtained from Shanghai SLAC Laboratory Animal. VX2 tumor mass (Shanghai Lalan Biotechnology, Shanghai, China) was grafted in the hind leg of the rabbit. After 15 days, the active VX2 tumor was obtained and cut into 1 mm^3^ pieces to implant into the left liver lobe of the rabbit for orthotopic models. For all orthotopic models, the tumors were confirmed using a 3.0 T magnetic resonance imaging (MRI Magnetom Skyra scanner (Siemens Healthcare GmbH, Erlangen, Germany).

### Interventional embolization

The procedure of N1S1 rat syngeneic orthotopic HCC model intervention was as follows. After opening the abdominal cavity, the hepatic and gastroduodenal arteries were identified and carefully dissected. Then, two ligatures were placed around the gastroduodenal artery and the distal part of the gastroduodenal artery was also ligated. A ligature was placed around the celiac artery to temporarily interrupt blood flow. The gastroduodenal artery was punctured upstream of the distal ligature using a self-made needle and then a catheter was placed into the hepatic artery. After the administration of the chemoembolization drug (4 mg/mL, 100 μL), the proximal part of the gastroduodenal artery (upstream of the puncture point) was tied off. The ligature around the celiac artery was then removed and hepatic arterial flow was restored (Figure S1). Afterward, the evaluation of the intervention was performed by computed tomography (CT) plain scan. The procedure of the VX2 rabbit syngenic orthotopic HCC model intervention was as follows. When the orthotopic VX2 tumor size reaches about 10 × 10 mm, the rabbits were subjected to the intervention using a percutaneous intra-arterial femoral port-catheter system with the guidance of digital subtraction angiography (DSA), and then the chemoembolization drug (200 μL, 3 mg/mL) was slowly injected into lesions. After that, the evaluation of the intervention was performed by CT plain scan.

### Magnetic resonance imaging monitoring

All rats (on 0, 3, 7 and 14 days) and rabbits (on 0, 5 and 10 days) from each group underwent MRI scanning using a Magnetom Skyra MRI scanner (Siemens Healthcare GmbH) to record the tumor size. All animals were under mild anesthesia to obtain stable and accurate images. The tumor size was estimated by its largest (L) and smallest (S) diameters using the following formula: Tumor volume/mm^3^ = (L × S^2^)/2.

### Histology and immunohistochemistry analysis

All rats were euthanized on day 14, and rabbits on day 10. Hearts, normal liver, spleen, lung and kidney tissues, and tumors in the liver were individually harvested. The samples were immediately immersed in 4% paraformaldehyde and embedded in paraffin. Rat tumor specimens were stained with hematoxylin and eosin (H&E), Ki-67 antibody and oil red. The rabbit samples were stained with H&E, Ki-67 antibody, terminal deoxynucleotidyl transferase dUTP nick end labeling (TUNEL), and the nuclei were stained with DAPI. All tumor tissue sections were examined by fluorescence microscopy with DOX. Tumor-bearing tissues were characterized by epithelial cellular shape and trabecular growth pattern 12. All histology samples were analyzed by pathology researchers with 10 years of experience.

## Results

### Preparation and characterization of nanoDOX *via* SPFT

**Figure 2:**
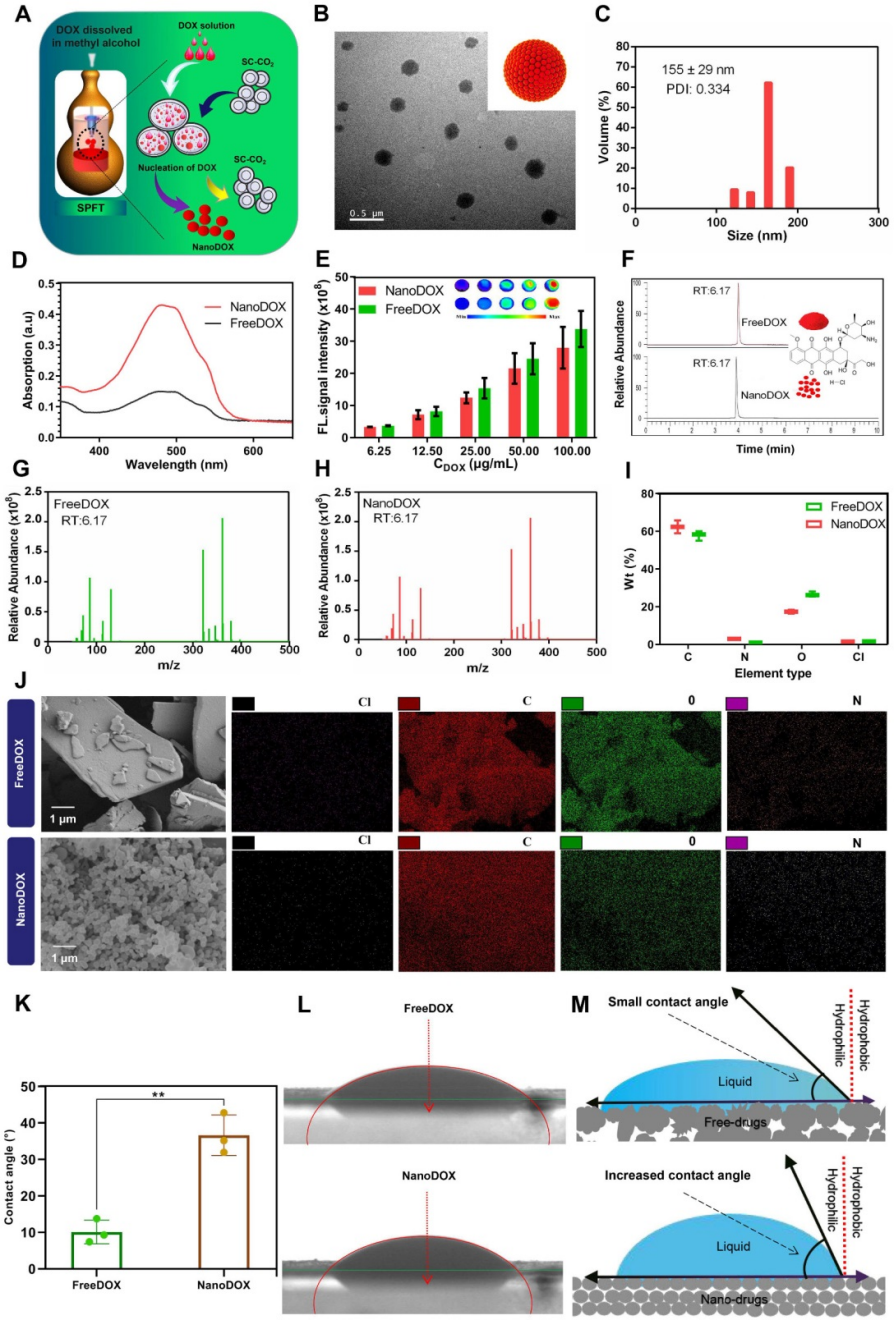
** Characterization of nanoDOX.** (**A**) Preparation of nanoDOX *via* SPFT. (**B**) Representative TEM image of nanoDOX. Scale bar: 0.5 μm. (**C**) DLS of nanoDOX. (**D**) FreeDOX and nanoDOX ultraviolet absorption spectra. (**E**) Fluorescent signal of freeDOX and nanoDOX at different concentrations. (**F, G, H**) The molecule structure of LC-MS of freeDOX and nanoDOX. (**I, J**) SEM and mapping of freeDOX and nanoDOX. (**K, L**) The contact angle of freeDOX and nanoDOX. (**M**) The proposed mechanism underlying the changed contact angle chart of freeDOX and nanoDOX. Data represent mean ± SD, n = 3. ** p < 0.01, Student's t-test.

A physically pure drug nanomedicine technology was applied to prepare nanoDOX. In detail, DOX was dissolved into the low-boiling point solvent methyl alcohol, and then the mixed solution was slowly transferred to a SC-CO_2_ reactor. CO_2_ was pumped into the reactor at a constant flow rate of 35 g/min to remove the solvent, resulting in the precipitation of homogeneous DOX pure nanoparticles (Figure 2A). TEM showed that nanoDOX nanoparticles displayed a spherical structure (Figure 2B). DLS analysis confirmed that the hydrated particle size of nanoDOX was 155 ± 29 nm (Figure 2C), indicating that nanoDOX has excellent application potential in biomedicine.

In addition, evaluation of the ultraviolet (UV) absorption property and optical property of nanoDOX and freeDOX revealed that the absorption (Figure 2D) and fluorescent imaging (Figure 2E) of nanoDOX had no obvious difference versus freeDOX. Furthermore, the determination of the molecular structure of freeDOX and nanoDOX by LC-MS indicated consistent molecular composition for nanoDOX versus freeDOX standard samples (Figure 2F-H). SEM analysis showed that the freeDOX consisted of large and heterogeneous aggregates, while nanoDOX consisted of homogeneous nanoparticles. The elemental composition in freeDOX and nanoDOX was consistent (Figure 2I-J), suggesting that the basic properties of DOX were not changed after nanocrystallization. The solid-liquid interface contact angle analysis showed that the contact angle of two different forms of drugs was less than 90 degrees, but the contact angle of nanoDOX increased by three times than that of freeDOX (Figure 2K-L). These data show that compared with freeDOX, spherical nanoDOX with a small and uniform size have enhanced hydrophobic property, suggesting a potential of stable dispersibility in lipiodol (Figure 2M) 24.

### Synthesis and characteristics of SHIFT&DOX formulation

**Figure 3:**
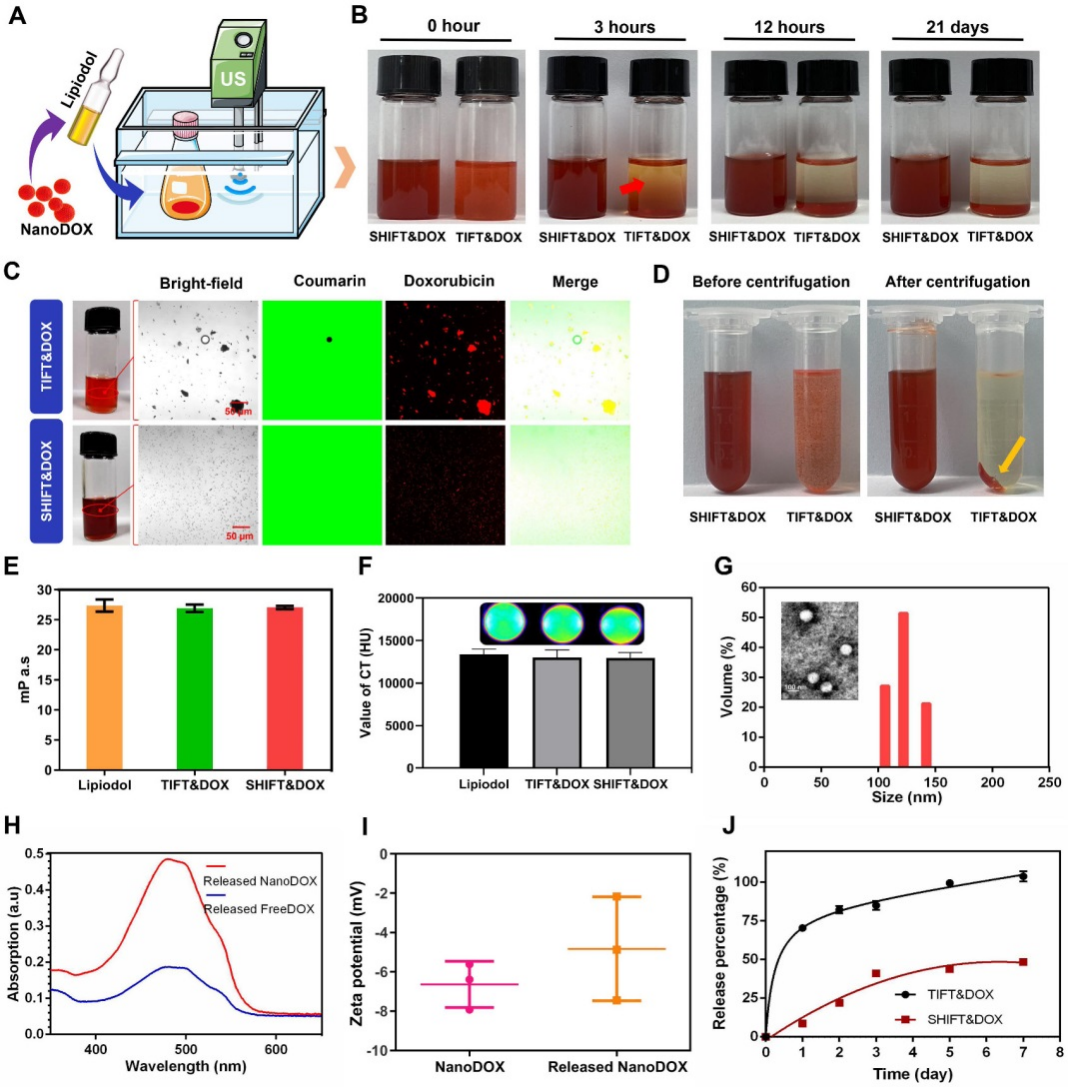
** Preparation and characterization of SHIFT&DOX.** (**A**) Schematic of SHIFT&DOX prepared *via* ultrasonication. (**B**) Photograph of a mixture of SHIFT&DOX and TIFT&DOX newly prepared and stored for 3 h, 12 h, and 21 days. (**C**) Confocal microscope image of freeDOX and nanoDOX scattered in coumarin-labeled lipiodol. (**D**) Photograph of the centrifuged formulation. (**E**) Viscosity of lipiodol, TIFT&DOX, and SHIFT&DOX. (**F**) CT of lipiodol, TIFT&DOX, and SHIFT&DOX. (**G**) Representative TEM image of released nanoDOX (Inset: DLS of released nanoDOX). (**H**) Ultraviolet absorption spectra of released freeDOX and nanoDOX. (**I**) Charge of nanoDOX and released nanoDOX. (**J**) Drug release curve of freeDOX and nanoDOX for 7 days.

Further, nanoDOX was blended homogeneously into lipiodol *via* ultrasonication to produce a super-stable homogeneous Lipiodol-DOX compound (SHIFT&DOX) (Figure 3A). Figure 3B shows that SHIFT&DOX is a clear and homogeneous red liquid suspension. No delamination or sediment was observed in the SHIFT&DOX formulation after 21 days standing at 25°C. The TIFT&DOX, which is commonly used in clinical TACE, is heterogeneous and unstable, thus quickly separates into two layers (within 30 min) at 25°C. The stability of the DOX in lipiodol was evaluated by confocal microscopy using coumarin-labeled lipiodol. The results suggest that freeDOX was unevenly distributed in TIFT&DOX as large and irregularly agglomerated particles in the lipiodol. In contrast, nanoDOX was evenly distributed in the lipiodol with uniform particles in SHIFT&DOX (Figure 3C). With a small particle size and regular morphology, nanoDOX increases the contact angle with lipiodol and reduces the gravity caused by agglomeration, thus preventing separation and sedimentation for a long time 24,25,27.

To further verify the stability of the formulation, we centrifuged SHIFT&DOX, and TIFT&DOX at 5,000 rpm for 5 min. After centrifugation, nanoDOX remained uniformly and steadily dispersed in the lipiodol, whereas freeDOX was deposited at the bottom of the centrifuge tube (Figure 3D). Maintaining viscosity and CT imaging performance is crucial for lipiodol to achieve effective embolization and monitoring. The comparison of the viscosity and CT value of SHIFT&DOX, TIFT&DOX and lipiodol revealed no obvious changes (Figure 3E-F). We next studied the state of nanoDOX in SHIFT&DOX, TEM showed that the nano-size was retained but the diameter decreased to 132 ± 14 nm (Figure 3G). UV absorption analysis of released drug from nanoDOX showed no obvious difference versus released freeDOX (Figure 3H), and both of them are negative with no obvious difference (Figure 3I). The release rate of TIFT&DOX was clearly faster than SHIFT&DOX in saline. Moreover, while nearly 100% of TIFT&DOX was released, only about 50% of SHIFT&DOX was released in 7 days, implying that SHIFT&DOX has good slow-release properties (Figure 3J, Figure S2).

### Evaluation of the DOX release behavior of SHIFT&DOX in a decellularized liver model

**Figure 4:**
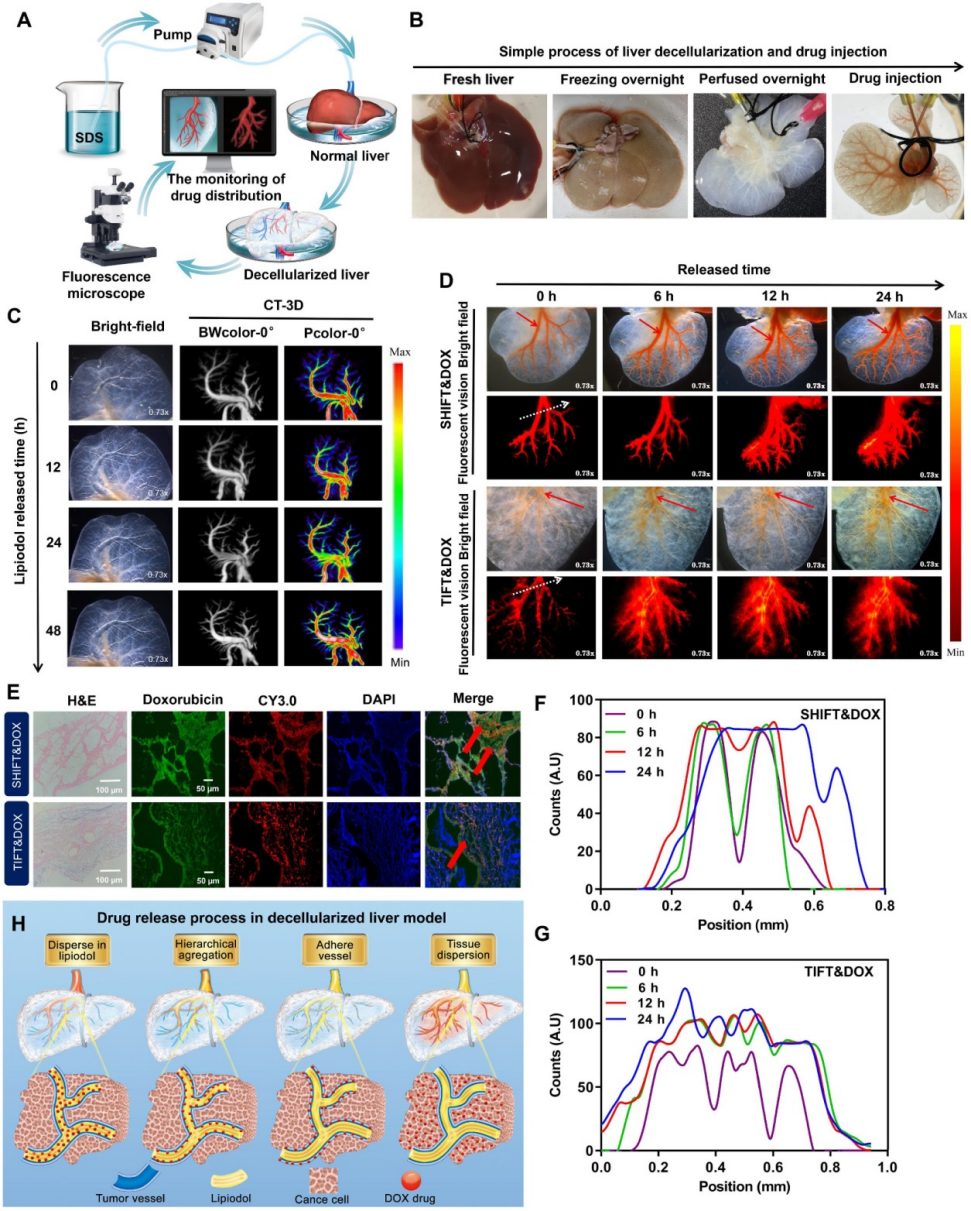
** Preparation and characterization of decellularized liver drug release model.** (**A, B**) The process of making decellularized liver model. (**C**) Representative 3D-CT images of lipiodol in a venous decellularized liver model within 24 h. (**D**) Representative SFM images of DOX released from SHIFT&DOX and TIFT&DOX of newly injected as well as samples stored for 6 h, 12 h, and 24 h. (**E**) Confocal microscopy image of tissue sections at 24 h. (**F, G**) Semi-quantitative analysis of fluorescence intensity at each time point based on the location of 0 h fluorescence (white dotted line). (**H**) Suggested flow chart of DOX drugs release behavior into blood vessels.

The properties of stable, homogenous, and slow drug release behavior of SHIFT&DOX were further investigated in a decellularized liver model. The decellularized liver model was verified by stereo fluorescence microscopy (SFM) and three-dimensional (3D)-CT (Figure 4A-B). The results indicated that the injected pure water-soluble freeDOX and nanoDOX into the decellularized liver venous completely diffused into the liver in 30 min (Figure S3), while the injected lipiodol remained stable for 24 h (Figure 4C). Distribution and drug release behavior of TIFT&DOX and SHIFT&DOX were evaluated after injection into the venous system of the decellularized liver model.

The results showed that TIFT&DOX could be dispersed into tiny blood vessels, but clustered at 0 h. FreeDOX was almost completely released from lipiodol within 6 h, and was completely released outside of blood vessels within 24 h (Figure 4D-G, S4). However, the SHIFT&DOX particles were evenly distributed and displayed a uniform morphology in decellularized liver, only a small amount of nanoDOX were released in 24 h (Figure 4D-F, S4). These results also confirmed by 24 h confocal microscopy imaging of tissue sections (Figure 4E). Accordingly, we deduce the release process of SHIFT&DOX is that nanoDOX is homogeneously dispersed in lipiodol, then accumulates in the blood vessel wall, finally crossed the blood vessel wall and diffused throughout the liver (Figure 4H). These results suggested that SHIFT&DOX has a better performance in stability, dispersibility, and sustained drug release.

### Cellular drug uptake and cytotoxicity of nanoDOX

**Figure 5:**
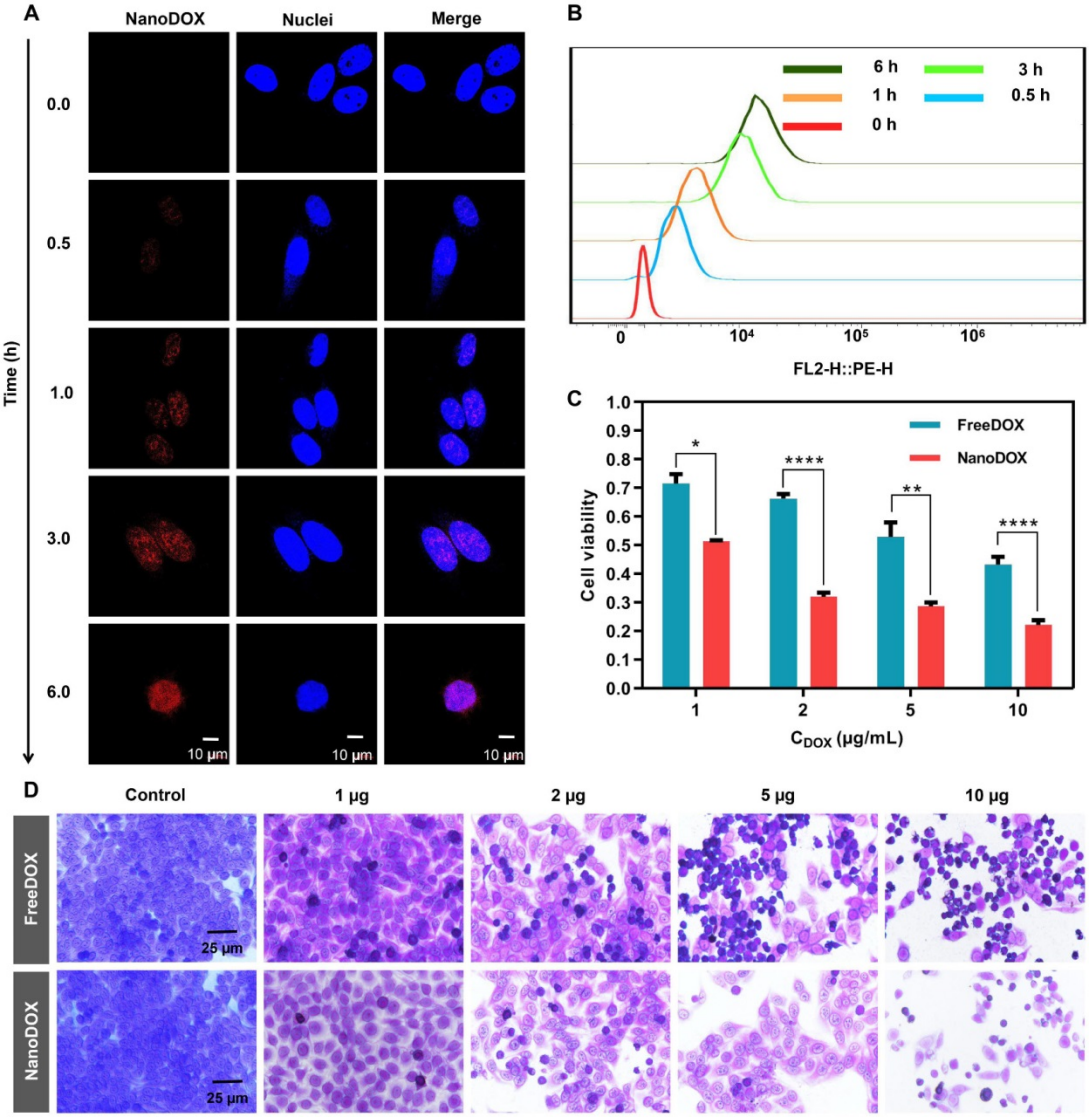
** Cellular uptake and cytotoxicity of nanoDOX. (A)** Confocal microscope imaging of HepG-2 cell uptake from nanoDOX with different time points. (**B**) Semi-quantitative analysis of HepG-2 cell uptake from nanoDOX with different time points. (**C**) Semi-quantitative analysis of cell viability of HepG-2 cells after 6 h incubation. (**D**) Crystal violet staining of HepG-2 cells after 6 h incubation. Data represent mean ± SD, n = 4. * p < 0.05; ** p < 0.01; **** p < 0.0001, Student's t-test.

DOX is a well-known chemotherapeutic drug that intercalates between deoxyribonucleic acid (DNA) base pairs, thus inhibiting DNA synthesis and transcription 28. We reasoned that nanoDOX released from SHIFT&DOX could have a better anti-tumor effect 29. To confirm cellular entry and nuclear accumulation of nanoDOX, we treated HepG-2 cells with nanoDOX for different times. After fixing cells with 4% paraformaldehyde and staining the nuclei with DAPI, confocal microscopy imaging showed that nanoDOX fluorescence (red) co-located with DAPI in the nucleus within 1 h. At 6 hours, nanoDOX entered the nucleus of tumor cells with large quantities (Figure 5A-B). Cytotoxicity was monitored with different concentrations of nanoDOX (1, 2, 5, 10 µg/mL) for 6 h incubation with HepG-2 cells using a water-soluble tetrazolium (WST) cell viability assay and crystal violet staining (Figure 5C-D). The results showed that nanoDOX is more toxic to HepG-2 cells than freeDOX, indicating a better tumor therapeutic efficacy of nanoDOX 29.

### Evaluation of treatment safety and efficacy of nanoDOX and SHIFT&DOX in N1S1 rat orthotopic HCC model

Drug safety is one of the main factors that influence clinical HCC treatment decisions. The toxicity of DOX mainly includes cardiac toxicity, renal toxicity and damage to liver function 30,31. In this study, the toxicity test of nanoDOX was evaluated *via* double (2 mg) and quadruple (4 mg) doses of freeDOX in specific pathogen-free SD rats (100 - 120 g) by serum biochemical analysis at 3 and 7 days post-injection with histological examinations of heart, liver, spleen, lung and kidney at seven days post-injection. The serum biochemical analysis results showed that the levels of alanine transaminase (ALT), aspartate transaminase (AST) and creatine kinase myocardial ban (CK-MB) in nanoDOX and freeDOX groups at 3 days post-injection were higher than blank group, but the levels in both groups were within the normal range 30. Specially, levels in the nanoDOX group were lower than those in the freeDOX group (Figure S5). However, there were no significant differences in the levels of ALT, AST, and serum-creatinine (S-CREA), CK-MB at 7 days post-injection with double doses (Figure S6). At a four-fold dose, ALT, AST, and CK-MB levels were higher than those in the blank group on 3 and 7 days post-injection, but they were within the acceptable range (2.7-fold). These levels were decreased by day 7 post-injection (Figure S7-8). H&E staining of heart, liver, spleen, lung, and kidney tissues showed no significant organ damage on day 7 (Figure S9). These findings indicate that nanoDOX prepared by SPFT is safe and has a high potential for clinical applications.

**Figure 6:**
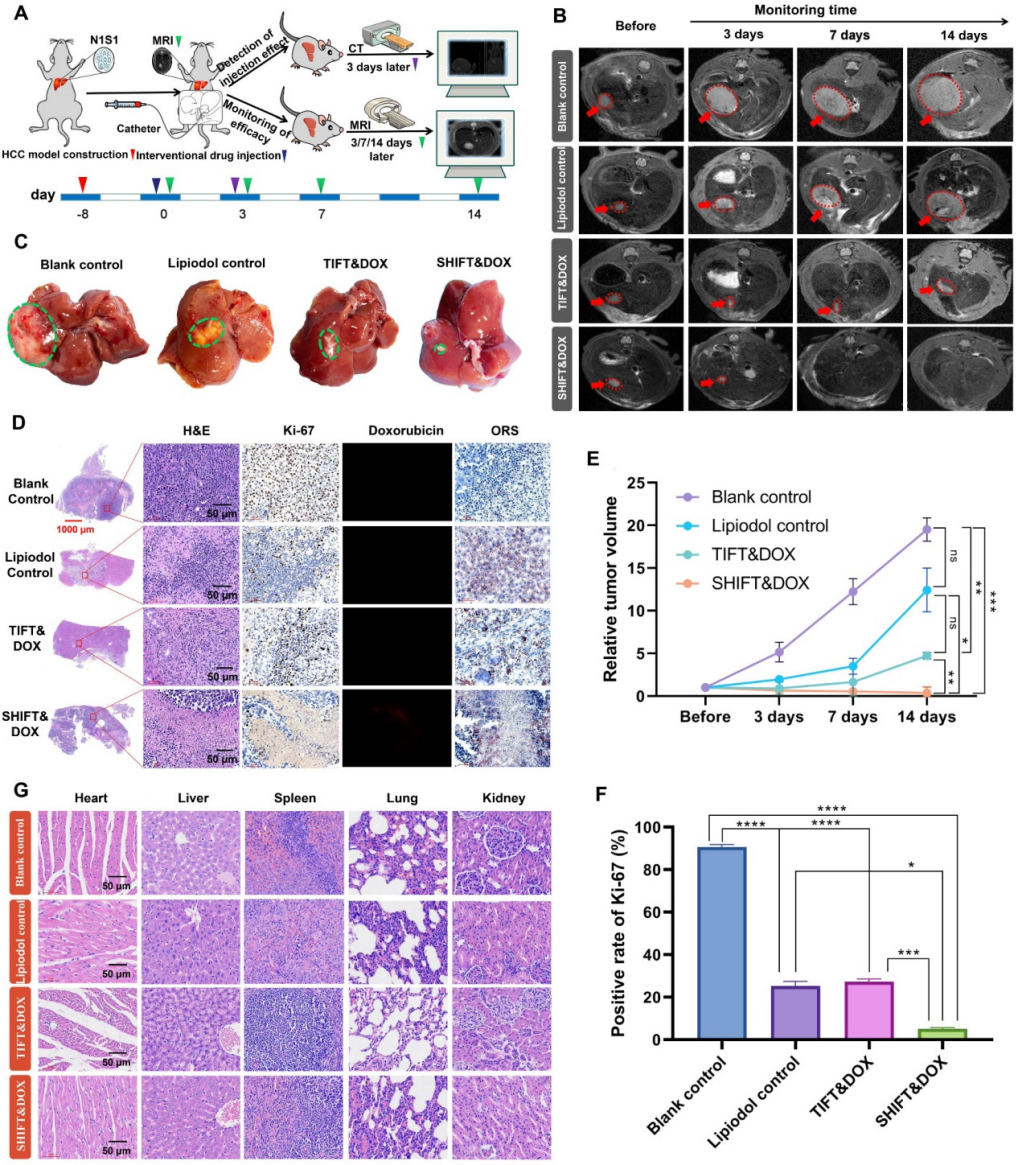
** Treatment efficacy of SHIFT&DOX in a rat orthotopic HCC model. (A)** Schematic of the evaluation process. (**B**) Representative MRI images. (**C**) Representative resected tumor lesions for 14 days. (**D**) Histological staining of representative resected tumor lesions for 14 days. (**E**) Quantitative analysis of relative tumor volume. (**F**) Quantitative analysis of Ki-67 positive rate for 14 days in tumors. (**G**) Tissue and organ safety evaluation. Data represent mean ± SD, n = 3. * p < 0.05; ** p < 0.01; *** p < 0.001, **** p < 0.0001, one-way ANOVA test.

Further evaluation of SHIFT&DOX was conducted in N1S1 rat syngeneic orthotopic HCC model. Figure 6A shows the N1S1 tumor orthotopic model developed in rat liver. After 8 days, the rat models were randomly divided into four groups: blank control, lipiodol control, TIFT&DOX, and SHIFT&DOX groups. Drugs (4 mg/mL, 100 μL) were accurately embolized into the hepatic artery under the guidance of DSA. CT monitoring was conducted (for 3 days) to assess lipiodol deposition in the HCC lesions (Figure S1). MRI was applied to monitor the treatment efficacy at 3, 7, and 14 days post-treatment. The MRI results revealed that SHIFT&DOX not only effectively inhibited tumor growth, but also reduced tumor size, and the tumors in two rats disappeared without recurrence (within 14 days). However, tumor growth of TIFT&DOX group was inhibited in the early stage after TACE, but tumor volume continued to increase in the later stage (14 days). Besides, the lipiodol group showed no obvious inhibitory effect on tumor growth (Figure 6B-C and E).

To further evaluate the therapeutic effects, we euthanized the rats with 14 days treatment and collected tumor tissues for histological analysis (Figure 6D). H&E staining results showed that the tumor of SHIFT&DOX group had a higher degree of tumor necrosis than other three groups. The tumor Ki-67 positive rate of SHIFT&DOX was 5.12 ± 0.62%, which was significantly lower than that of the other three groups (TIFT&DOX, lipiodol, and blank with Ki-67 positive rates of 27.28 ± 1.37, 25.39 ± 2.09, 90.58 ± 1.32 percent, respectively; Figure 6D, F). These results suggest that SHIFT&DOX acted as a better chemoembolization formulation. The histological analysis of DOX fluorescence and lipiodol oil red staining (ORS) also verified DOX and lipiodol were deposited in tumor lesions after 14 days treatment (Figure 6D). The remarkable fluorescent imaging (FLI) of the DOX in resected tumor lesions indicated that the increased DOX concentration in the tumor sites of SHIFT&DOX than that of TIFT&DOX group (Figure 6D). Safety evaluation results indicated no significant tissue damage in animals of the SHIFT&DOX group (Figure 6G). These findings suggested that nanoDOX in lipiodol could preserve drug functionality while slowly released.

### Evaluation of the treatment efficacy and safety of SHIFT&DOX in VX2 rabbit syngeneic orthotopic HCC model

**Figure 7:**
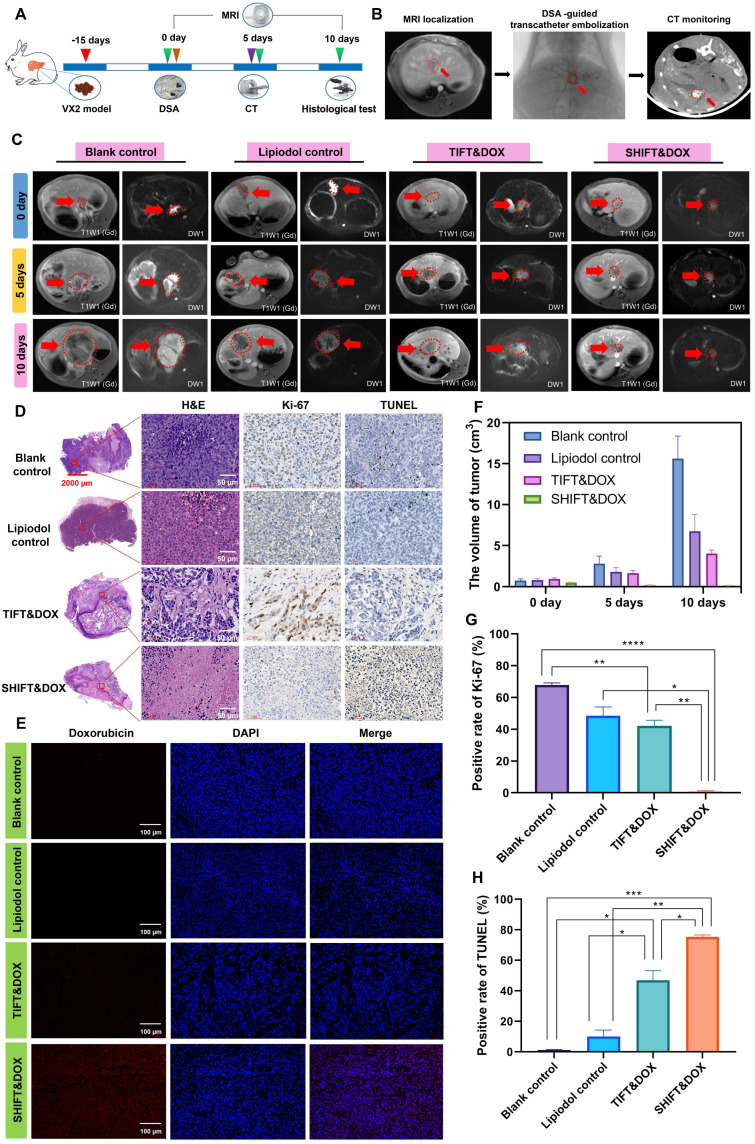
** Evaluation of treatment efficacy of SHIFT&DOX in rabbit orthotopic HCC models.** (**A**) Schematic of VX2 orthotopic models and treatments. (**B**) Verification of VX2 orthotopic models *via* MRI and DSA-guided embolization of SHIFT&DOX as well as CT-monitored embolic evaluation. (**C**) Representative MRI images. (**D**) Representative histological staining of resected tumor lesions after 10 days embolization. (**E**) Representative fluorescence images of DOX in resected tumor tissues. (**F**) Quantitative analysis of tumor volume. (**G, H**) Quantitative analysis of Ki-67 and TUNEL positive rate in tumor specimen after 10 days in tumors. Data represent mean ± SD, n = 3. * p < 0.05; ** p < 0.01; *** p < 0.001, **** p < 0.0001, one-way ANOVA test.

We next used the VX2 rabbit syngeneic orthotopic HCC model to evaluate the advantages of SHIFT&DOX in conditions mimicking TACE (Figure 7A). First, a rabbit VX2 primary syngeneic orthotopic HCC model was developed and confirmed by MRI. Rabbits were randomly divided into four groups: blank control, lipiodol control, TIFT&DOX, and SHIFT&DOX. TACE was performed *via* the femoral artery under the guidance of DSA, and CT was used to examine the lipiodol deposition in lesions on the fifth day after surgery (Figure 7B). The therapeutic effect and sustained drug release was evaluated by MRI on the 5th and 10th day after treatment. Tumor histological analysis was performed on the 10th day. The safety was evaluated by hematological and serum biochemical analysis at the 3, 7, and 10 days after treatment, and H&E histological examination of the heart, liver, spleen, lung, and kidney tissues at the 10 day after treatment. MRI results showed that SHIFT&DOX had significant tumor inhibition with large and deep tumor necrosis areas, but TIFT&DOX group showed weak tumor inhibition than blank and lipiodol control group. H&E staining results showed the tumors of SHIFT&DOX group had a higher degree of tumor necrosis than those in the other three groups. Immunohistochemistry (IHC) analysis revealed that the positive rates of Ki-67 staining in the SHIFT&DOX, TIFT&DOX, lipiodol control and blank groups were 0.79 ± 0.47, 44.89 ± 8.05, 48.55 ± 5.48, 67.94 ± 1.31%, respectively (Figure 7D, G), suggesting the significant inhibition of tumor proliferation with SHIFT&DOX treatment. Besides, the positive rate of TUNEL in the SHIFT&DOX group was significantly higher than that of the other three groups (Figure 7D, F). These data suggest that SHIFT&DOX had a better chemoembolization effects.

Fluorescence imaging of the resected tumor lesions showed that SHIFT&DOX group favored a higher DOX concentration. The FLI intensity of DOX could barely be seen in the TIFT&DOX group (Figure 7E) also suggesting that SHIFT is a reliable technique for slow release of DOX. In addition, safety was evaluated by H&E staining of the heart, liver, spleen, lung, and kidney tissues of the rabbit model: There was no observable damage 10 days after treatment (Figure S10). Moreover, the postoperative hematological and serum biochemical analysis results showed that the white blood cell (WBC), ALT, AST, S-CREA, and CK-MB levels in the lipiodol control, TIFT&DOX and SHIFT&DOX groups were higher than those in the blank group on the 3rd and 7th days after treatment (Figure S11-12). On the 10th days, the levels of those indicators were similar to those in the blank group (Figure S13). This finding indicates that SHIFT&DOX prepared by SHIFT is safe and reliable, and provides a basis for later clinical transformation.

## Discussion

Lipiodol-based TACE offers drug delivery and embolization to treat clinically inoperable patients 32-34. However, the traditional drug-lipiodol formulation is extremely unstable, and the sudden drug release leads to poor therapeutic effects, as well as some side effects 17. Therefore, the manufacture of hydrophilic drugs in lipiodol for sustained drug release is difficult. Herein, we designed a facile and economic method based on SPFT to make chemotherapeutic DOX presenting nanostructures (nanoDOX), which could be stably dispersed in lipiodol through a simple ultrasonication step. This method solved the drawbacks of traditional embolization formulations, such as poor stability and fast drug release. Moreover, it offered low systematic toxicity and better tumor local treatment of hydrophilic drugs. The principle of stable dispersion might be that nanoDOX could increase the contact angle with lipiodol *via* regularly spherical morphology and slight gravity to achieve a colloidal-like stability 24,25,27. Hence, SHIFT&DOX can provide a new, safe, efficient, and economical treatment for patients with advanced HCC.

The basis of SPFT is supersaturated precipitation and nucleation growth, which is a green physical process. Our results show that nanoDOX only has change in the morphology and without any molecular or molecular weight changes compared with freeDOX. Therefore, the function of nanoDOX is not affected and does not present any additional toxicity. Moreover, the prepared SHIFT&DOX exhibited excellent stability and dispersibility with excellent sustained slow-release effects, tumor treatment effects, and safety profiles. It is believed that SPFT can be applied to other chemotherapeutics 20 such as platinum and lenvatinib. Limitations include a lack of a detailed mechanism for stabilization of hydrophilic drugs with lipiodol after SPFT nanocrystallization. The impact on human patients also remains unknown. These issues are expected to be solved through digital modeling, fluid mechanics, and multicenter double-blind randomized controlled clinical trials. The focus of our next step is to solve these issues.

Notably, we have validated that SHIFT-indocyanine green formulations have excellent performance, safety, and effectiveness in a clinical trial for HCC resection (Register No. ChiCTR2000035055, data not shown). SHIFT&DOX also has excellent prospects for clinical translation, featuring clear components, sufficient physical stability, large-scale production, and maintaining the salient features pertinent to lipiodol. These overcome issues in clinical translation of the main nanoparticle production methods.

In summary, this facile and green SHIFT&DOX integrates excellent stability, homogeneity, excellent drug release behavior, specific tumoral deposition of lipiodol, and safety. To treat unresectable HCC, super-stable homogeneous lipiodol-hydrophilic drug formulations are needed with potential for clinical transformation and practical applications.

## Supplementary Material

Supplementary figures.

## Abbreviations

TACE: Transcatheter arterial chemoembolization
HCC: Hepatocellular carcinoma
DOX: Doxorubicin hydrochloride
SHIFT: Super-stable homogeneous intermixed formulation technology
SC-CO_2_: Supercritical carbon dioxide
ICG: Indocyanine green
SPFT: Superstable pure-nanomedicine formulation technology
US: Ultrasonication
TIFT: Traditional iodinated formulation technology
TEM: Transmission electron microscopy
HPLC: High-pressure liquid chromatography
SEM: Scanning electron microscopy
LC-MS: Liquid chromatography-mass spectrometry
DLS: Dynamic light scattering
UV: Ultraviolet
SDS: Sodium dodecyl sulfate
DAPI: 4′,6-diamidino-2-phenylindole
SD: Sprague-Dawley
DSA: Digital subtraction angiography
TUNEL: Terminal deoxynucleotidyl transferase dUTP nick end labeling
ORS: Oil red staining
SFM: Stereo fluorescence microscopy
ALT: Alanine transaminase
AST: Aspartate transaminase
S-CREA: Serum-creatinine
CK-MB: Creatine kinase myocardial ban
FLI: Fluorescent imaging
