# Supplementary material for: A super-stable homogeneous Lipiodol-hydrophilic chemodrug formulation for treatment of hepatocellular carcinoma
Source: Theranostics. 2022 Jan 24;12(4):1769–82. doi: 10.7150/thno.68456 (PMC8825598; doi:10.7150/thno.68456)
Supplement: Supplementary file 1 — Supplementary figures. [file thnov12p1769s1.pdf]

Supplementary Information

A superstable homogeneous Lipiodol-hydrophilic chemodrugs formulation for treatment of hepatocellular carcinoma

Pan He[1,2,#], En Ren[1,#], Biaoqi Chen[3,#], Hu Chen[1,#], Hongwei Cheng[1], Xing Gao[1], Xiaoliu Liang[1], Hao Liu[3], Jingdong Li[5], Bo Li[6], Aizheng Chen[3], Chengchao Chu[1,4], Xiaoyuan Chen[7], Jingsong Mao[1,2,*], Yang Zhang[1,*], Gang Liu[1,*]

[1] State Key Laboratory of Molecular Vaccinology and Molecular Diagnostics, Center for Molecular Imaging and Translational Medicine, School of Public Health, Xiamen University, Xiamen 361102, China.

[2] Department of Radiology, Xiang'an Hospital of Xiamen University, School of Medicine, Xiamen University, Xiamen 361102, China.

[3] Fujian Provincial Key Laboratory of Biochemical Technology, Institute of Biomaterials and Tissue Engineering, Huaqiao University, Xiamen 361021, China.

[4] Amoy Hopeful Biotechnology Co., Ltd., Xiamen 361027, China.

[5] Department of General Surgery, Institute of Hepatobiliary-Pancreatic-Intestinal Diseases, Affiliated Hospital of North Sichuan Medical College, Nanchong 637000, China.

[6] Department of General Surgery (Hepatobiliary Surgery), The Affiliated Hospital of Southwest Medical University, Luzhou 646000, China.

[7] Departments of Diagnostic Radiology, Chemical and Biomolecular Engineering, and Biomedical Engineering, Yong Loo Lin School of Medicine and Faculty of Engineering, National University of Singapore, Singapore, Singapore.

* Corresponding author. E-mail addresses: maojingsong163@163.com (J. Mao), zhangyang0823@xmu.edu.cn (Y. Zhang), gangliu.cmitm@xmu.edu.cn (G. Liu).

[#] P. He, E. Ren, B. Chen, and H. Chen contributed equally to this work.

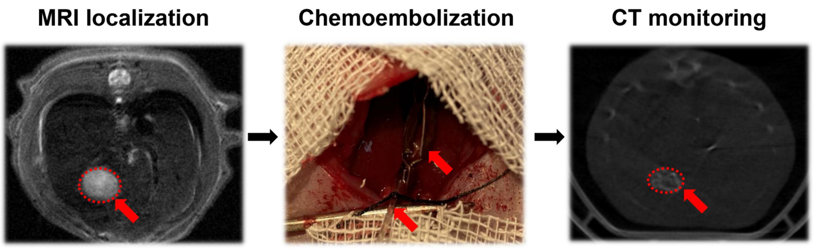

Figure S1. Verification of the N1S1 orthotopic models *via* MRI, embolization of SHIFT&DOX and CT monitored embolic evaluation.

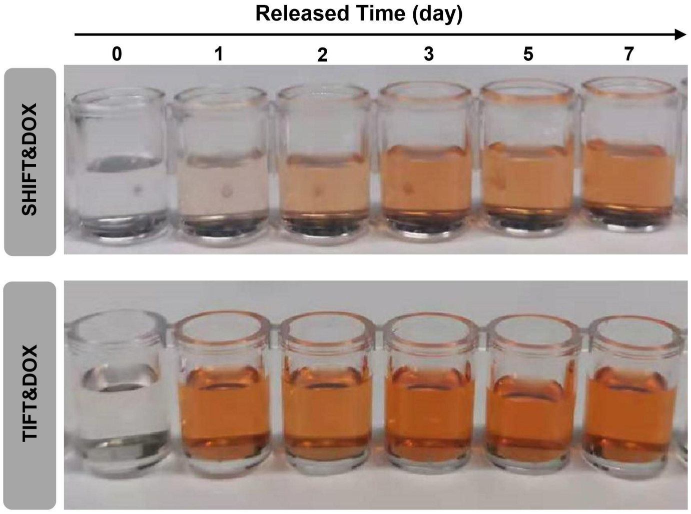

Figure S2. The colour photo of drugs release for freeDOX, nanoDOX within 7 days.

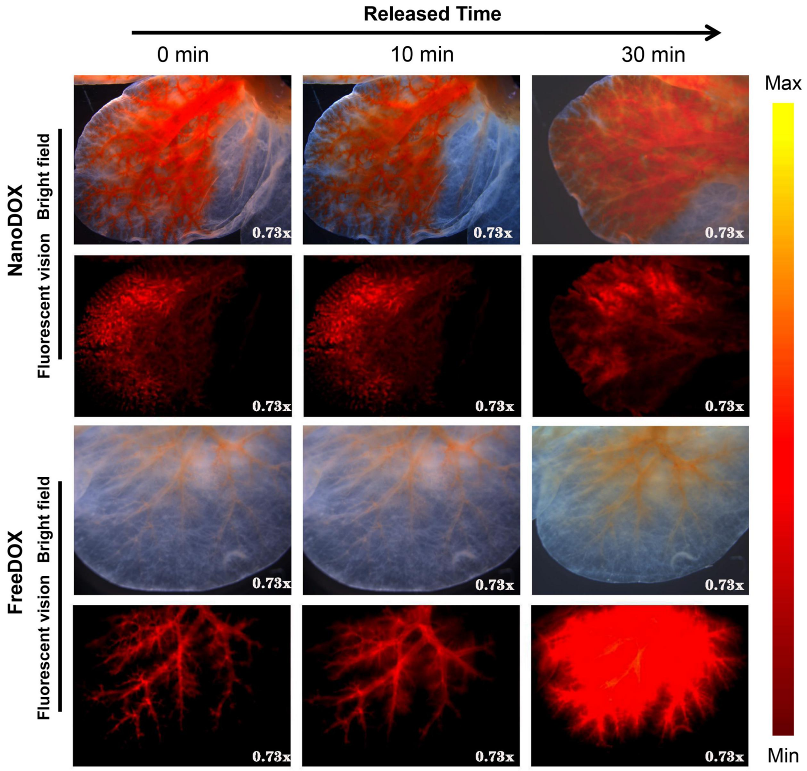

Figure S3. The typical fluorescence microscope images of pure water-soluble freeDOX and nanoDOX injection in the decellularized liver venous.

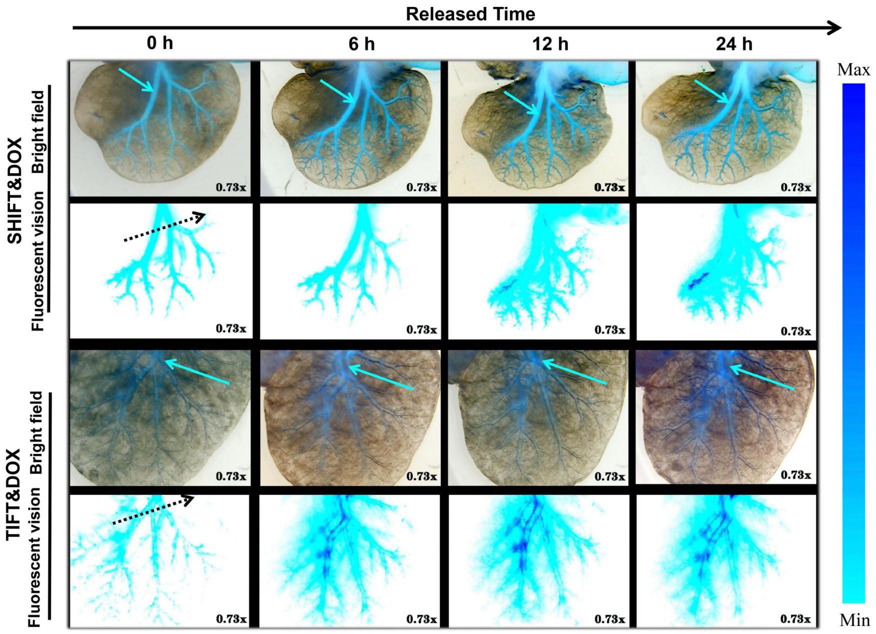

Figure S4. Representative SFM images with reverse phase (Adobe Photoshop CC 2019) of DOX released from SHIFT&DOX and TIFT&DOX of newly injected as well as samples stored for 6 h, 12 h, and 24 h.

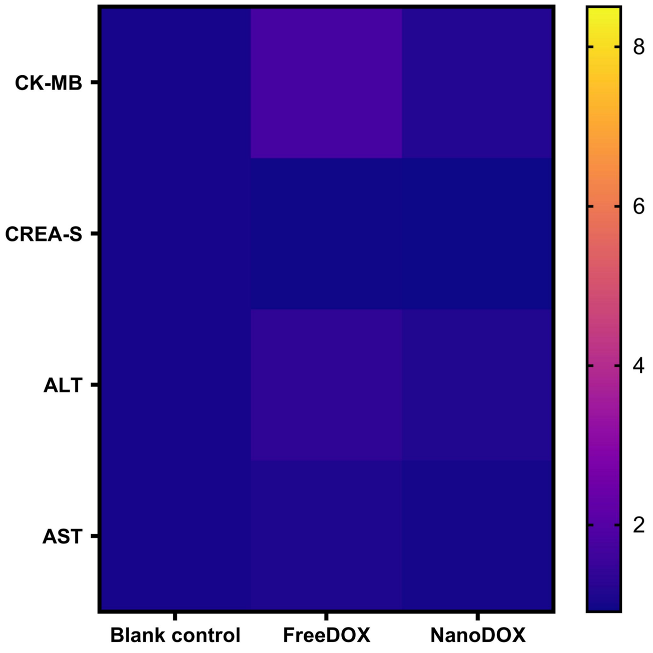

Figure S5. The biochemical analysis results of SD rat acute toxicity test on 3 days at the double doses (2 mg).

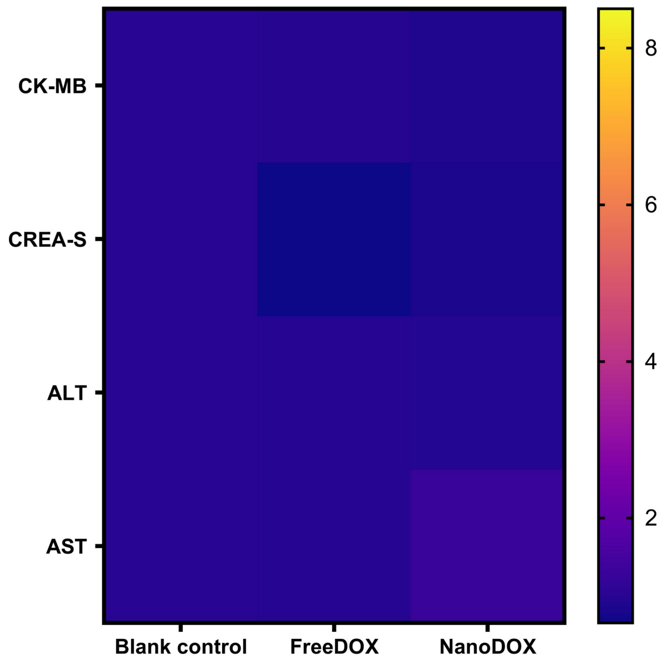

Figure S6. The biochemical analysis results of SD rat acute toxicity test on 7 days at the double doses (2 mg).

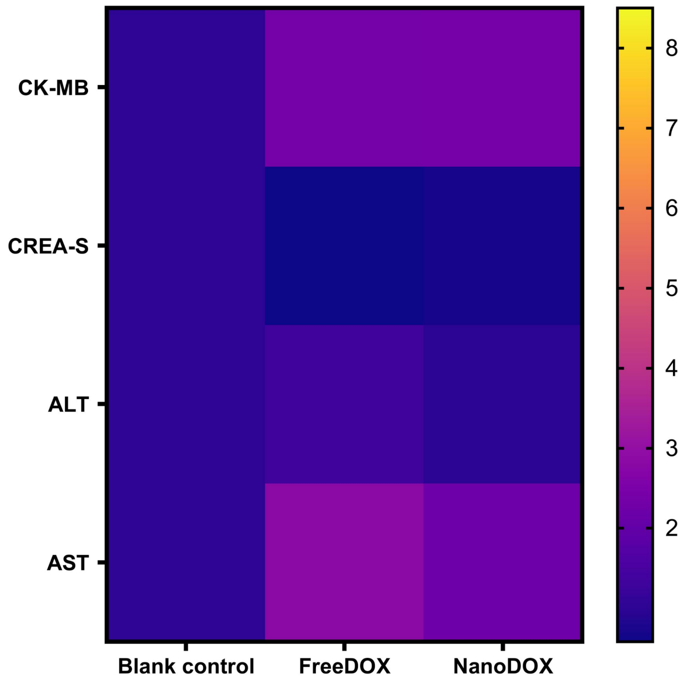

Figure S7. The biochemical analysis results of SD rat acute toxicity test on 3 days at the quadruple doses (4 mg).

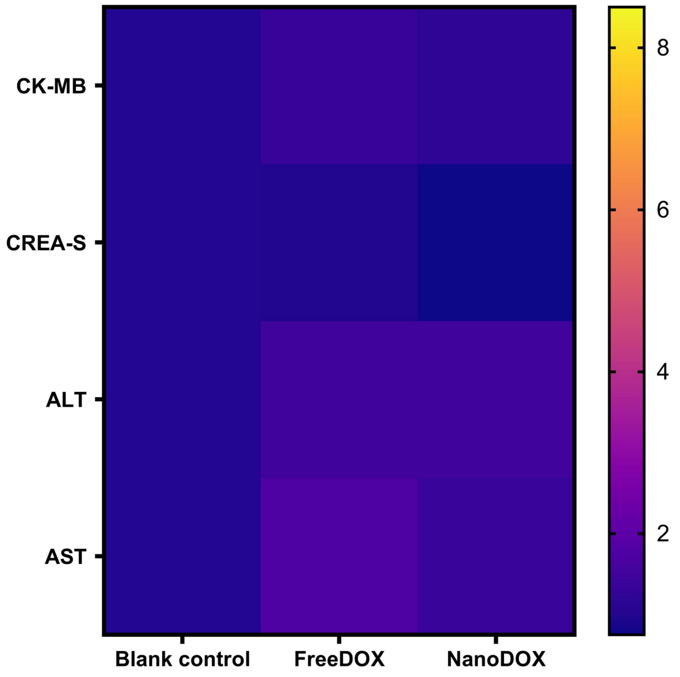

Figure S8. The biochemical analysis results of SD rat acute toxicity test on 7 days at the quadruple doses (4 mg).

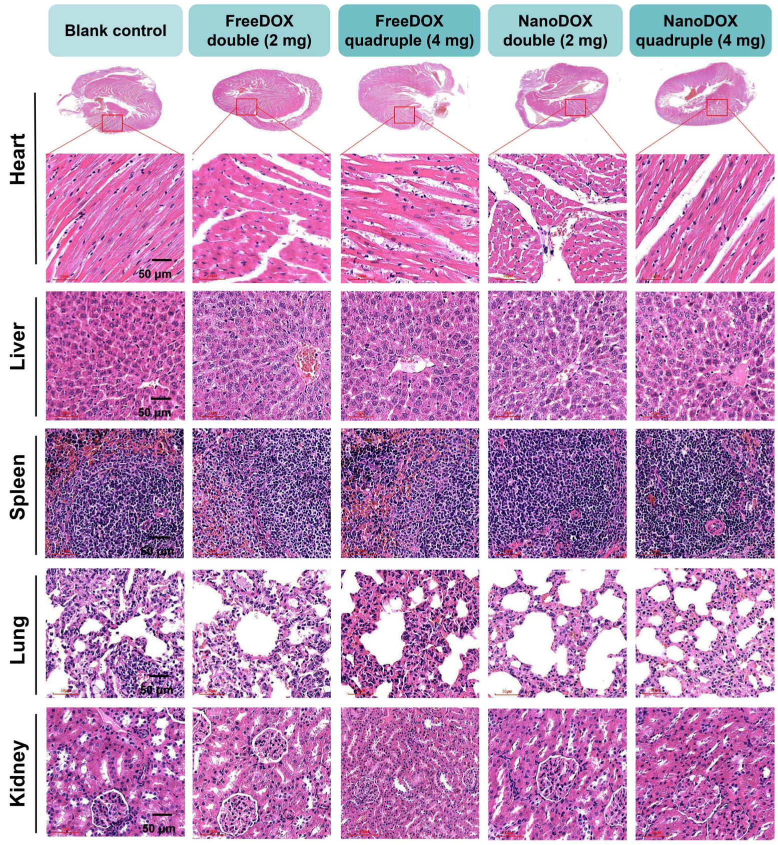

Figure S9. The H&E tissue staining of heart, liver, spleen, lung and kidney of SD rat acute toxicity test on 7 days after TACE.

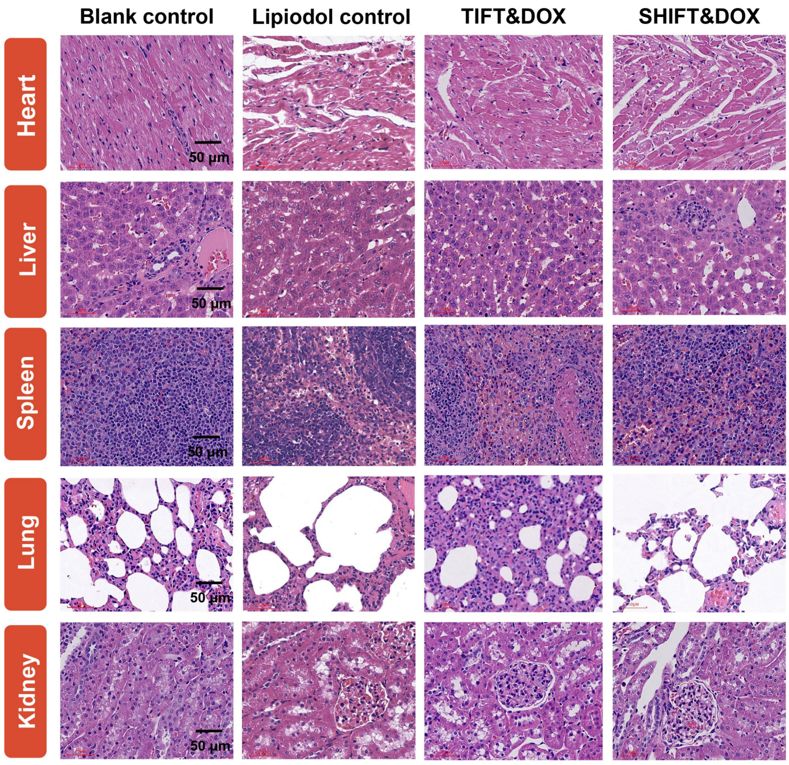

Figure S10. The H&E tissue staining of heart, liver, spleen, lung and kidney of the rabbit model on 10 days after TACE.

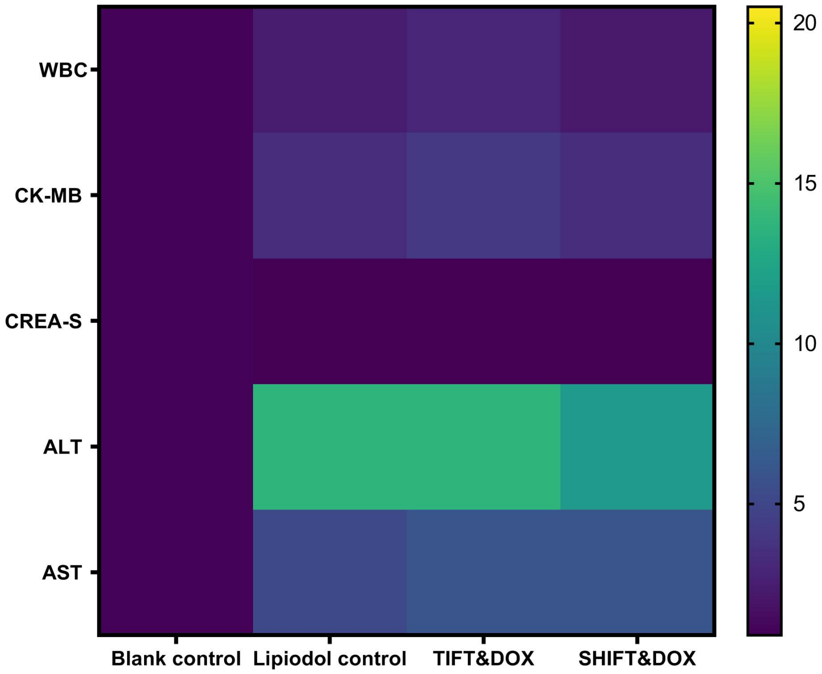

Figure S11. The blood cells and biochemical analysis results on 3 days after TACE.

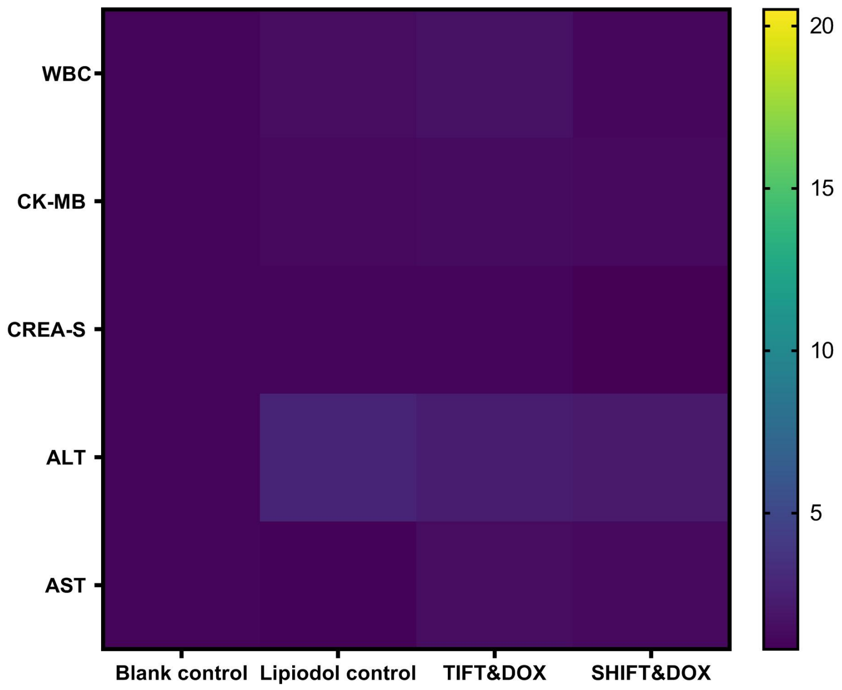

Figure S12. The blood cells and biochemical analysis results on 7 days after TACE.

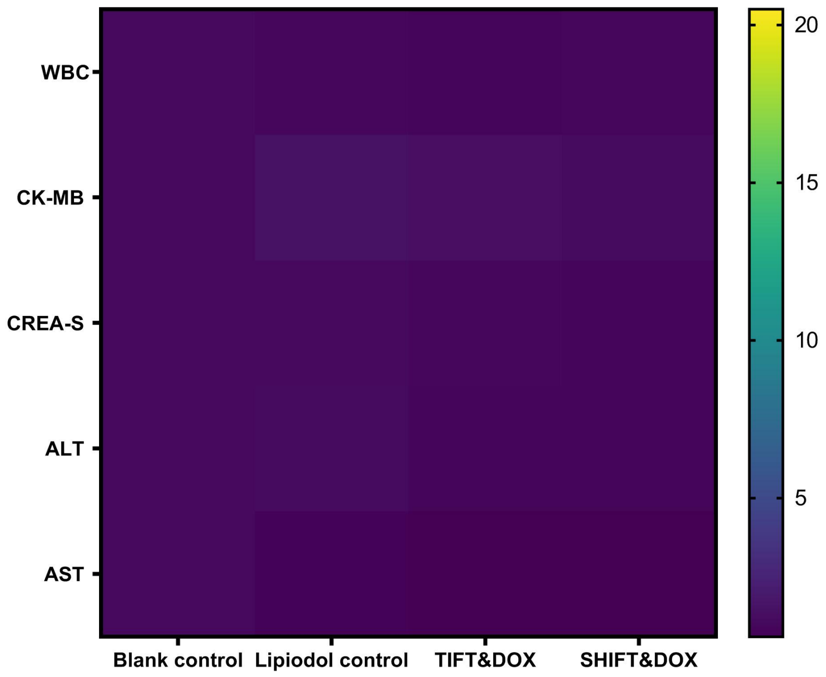

Figure S13. The blood cells and biochemical analysis results on 10 days after TACE.
